# Depression and related factors after oral oncological treatment: a 5-year prospective cohort study

**DOI:** 10.1007/s00520-020-05795-1

**Published:** 2020-10-01

**Authors:** Caroline M. Speksnijder, Petra J. M. Lankhorst, Remco de Bree, Anton F. J. de Haan, Ron Koole, Matthias A. W. Merkx

**Affiliations:** 1 Cancer Center, Department of Head and Neck Surgical Oncology, University Medical Center Utrecht, Utrecht University, Utrecht, The Netherlands; 2 Department of Oral and Maxillofacial Surgery, Radboud University Medical Center, Nijmegen, The Netherlands; 3 Department of Oral and Maxillofacial Surgery and Special Dental Care, University Medical Center Utrecht, Utrecht University, P.O. Box 85.500, 3508 GA Utrecht, The Netherlands; 4 Department of Medical Oncology, Deventer Hospital, Deventer, The Netherlands; 5 Department for Health Evidence, Section Biostatistics, Radboud University Medical Center, Nijmegen, The Netherlands

**Keywords:** Oral cancer, Head and neck cancer, Depression, CES-D, Coping style, CISS-21

## Abstract

**Purposes:**

Being diagnosed with oral cancer is a life-threatening life event. It often induces social, emotional and psychological consequences and may cause depressive disorders. The primary aim of this study was to identify and quantify the personal and clinical characteristics involved in depression for patients who have been treated for oral cavity malignancies, with a 5-year follow-up period after treatment. The secondary aim of this study was to identify the clinical factors that increase a patient’s risk of experiencing depression 5 years after treatment.

**Methods:**

Patients with primary oral cancer were assessed for up to 5 years after primary treatment. A mixed-model analysis was performed, with depression measured by the Center for Epidemiologic Studies Depression Scale as outcome measure.

**Results:**

A total of 141 patients were included in the study. Factors associated with depression were gender, tumour location and having an emotion-oriented coping style. The occurrence of depression within 5 years after treatment could be reliably predicted by a patient’s gender, the location of their tumour and the extent to which they had an emotion-oriented coping style.

**Conclusions:**

This study revealed that being female, having a maxillary tumour and having an emotion-oriented coping style are associated with higher levels of depressive symptoms in patients treated for oral cancer up to 5 years post-treatment. A substantial proportion of the patients with oral cancer experienced high levels of depression both before and after their treatment, suggesting that adequate diagnostics and care are needed to try to prevent severe depression in these patients.

## Introduction

Head and neck cancers are among the top ten most common causes of cancer-related deaths worldwide [1]. In the Netherlands, patients with oral cancer comprised of 28% of all patients with head and neck cancer [2]. Being diagnosed with oral cancer is a life-threatening life event. It often induces social, emotional and psychological consequences and may be associated with depressive disorders [3]. Depression is a common mental disorder in all patients with cancer [4]. The World Health Organization (WHO) defines depression as a disorder that lasts at least 2 weeks and is characterised by a person’s persistent sadness, loss of interest in activities that he or she normally enjoys and an inability to perform daily activities [4]. In 2011, a meta-analysis was conducted on 66 studies to determine the prevalence of depression in people with cancer, revealing that major depression was reported in 16.3% (95% CI: 13–20%) of these patients [5].

The oncological curative treatment of early-stage oral tumours usually involves surgery on indication, followed by radiotherapy and/or chemotherapy; however, these treatments and the resulting oral function deficits are associated with depression rates of 18% to 41% in patients treated for oral cancer [6–10]. A patient’s diet and ability to speak are often affected by these treatments [11]. Depression is a risk marker for a long-term reduced quality of life (QoL) in patients with oral cancer [12]. Previous studies have shown that personal and clinical characteristics, such as gender, age [13], marital status [14], education level, alcohol consumption [15], smoking behaviour [16], cancer stage [14] and cancer location [17], are factors associated with depression. Individual psychosocial characteristics, such as personality traits and coping styles, are also related to the depressive symptoms of patients with oral cancer [18–20].

To pursue an optimally targeted depression intervention, it is important to identity the risk factors for depression in patients with oral cancer [21–26]. The existing prospective cohort studies have a maximum follow-up of 1 to 3 years [17, 19, 27, 28]; therefore, the primary aim of this study was to identify and quantify the personal and clinical characteristics involved in depression for patients who have been treated for oral cavity malignancies, with a 5-year follow-up period after treatment. The secondary aim of this study was to identify the clinical factors that increase a patient’s risk of experiencing depression 5 years after treatment. The tertiary aim was to identify and quantify the personal characteristics involved in depression in healthy persons.

## Methods

### Subjects

In this multi-centre prospective cohort research, the study population consisted of patients with a primary malignant tumour involving the oral cavity who were referred to the University Medical Center Utrecht (UMCU) or Radboud University Medical Center (Radboudumc) between January 2007 and August 2009. Patients were recruited at these medical centres before the oral oncological intervention. The eligibility criteria were the presence of primary malignant tumours involving the oral cavity. The exclusion criteria included a previous and/or current second primary malignancy, cognitive impairment and the inability to understand Dutch. Sixty healthy age-matched controls were measured once and recruited at the UMCU and 3 dental practices, whose details were published previously [29]. The study protocol (study ID: NL1200604106) was approved by the Ethics Committees of the UMCU and Radboudumc. All participants received written information with details regarding the study and provided their signed informed consent. All measurements took place at the place of recruitment (i.e. UMCU, Radboudumc or dental practice).

Radiotherapy was given in accordance with the Dutch Head and Neck Society treatment guidelines, with a total radiation dosage of 64 to 70 Gy. Adjuvant radiotherapy, when given, started within 4 to 6 weeks after surgery.

The tumour locations of the oral cancers included the codes C00, C02 to C06 and C31 of the WHO International Classification of Diseases Oncology third edition (WHO ICD-0-3) [30]. Maxillary tumours included those on the upper alveolar process, tuber maxillae, palate and maxillary sinus (C03.0, C05, C31.0). Mandibular tumours included those on the lower alveolar process, the retromolar trigonum, the buccal mucosa and the lower lip (C00.4, C03.1, C06.0, C06.1, C06.2). Tongue and floor-of-the-mouth tumours included those located on the tongue and the anterior floor of the mouth (C02, C04) [30].

### Assessments

Patients were assessed by filling in the Center for Epidemiologic Studies Depression Scale (CES-D) at a maximum of 4 weeks before their primary treatment (baseline, *t*_0_); at 4 to 6 weeks after surgery (*t*_1a_) and/or 4 to 6 weeks after radiotherapy (*t*_1b_); and at 6 months (*t*_2_), 1 year (*t*_3_) and 5 years (*t*_4_) after their primary treatment.

Patient information, including gender, age, tumour location (maxilla, mandibula, tongue and/or floor of the mouth) and size (pT of TNM classification), resection site and details of reconstruction, were extracted from medical records. A maximum of 4 weeks before their primary treatment (baseline, *t*_0_) body mass index (BMI; in kg/m^2^), education level (primary education, secondary education, bachelor degree or master degree), living situation (living alone with or without children, with a partner with or without children), marital status (married or living together, unmarried, divorced or widowed), occupational status (working or not working), smoking status (current, former or non-smoker) and alcohol consumption (less than one unit per day, two to four units per day or more than five units per day) were charted by a questionnaire on paper. Patient coping styles were assessed by filling in the Coping Inventory for Stressful Situations-21 (CISS-21) on paper at *t*_0_.

### Center for Epidemiologic Studies Depression Scale

The main endpoint was the depression scores, which were determined using a questionnaire. At every assessment, the depressive symptoms were evaluated. The CES-D is a self-assessment questionnaire comprising 20 items [31]. Each item was scored on a 4-point Likert scale from seldom/never (0) to mostly/always (3), generating a range of 0 to 60 points in total. A higher score indicates a higher level of depression, with a CES-D score of 16 or more being considered a rough indicator of clinical depression in patients with oral cavity malignancies [32, 33]. This rough indicator has not been completely validated but can be used as a cut-off point for clinical depression [34]. The CES-D has a good internal consistency, with a Cronbach’s alpha score between 0.79 and 0.92 [34].

### Coping Inventory for Stressful Situations-21

The CISS-21 questionnaire comprises seven items on task orientation, seven items on emotional orientation and seven items on avoidance coping [35]. Task-oriented coping refers to purposeful task-oriented efforts aimed at solving the problem, cognitively restructuring the problem or attempting to alter the situation. The emphasis is on the task or planning and attempts to solve the problem. Emotion-oriented coping refers to self-oriented emotional reactions, which include emotional responses, self-preoccupation and fantasising. Avoidance coping refers to activities and cognitive changes aimed at avoiding the stressful situation by distracting oneself with other situations or tasks, or via social diversion as a means of alleviating stress. Every item was scored on a 5-point Likert scale ranging from not at all (0) to very often (4), with a score range of 0 to 28 per coping strategy. The higher the score for a coping strategy, the more dominant that coping strategy is in that person. CISS-21 is a shorter version of the original CISS questionnaire, which comprises 48 items and has been validated. The CISS-48 questionnaire has an internal consistency between 0.66 and 0.81 and a reliability between 0.65 and 0.78 [35]. The coping styles were evaluated at *t*_0_ in this study.

### Statistical analysis

Categorical patient characteristics are presented as numbers and percentages, while continuous characteristics are presented as means and standard deviations in the case of normally distributed variables. A mean imputation of the missing values was performed with continuous data and median with ordinal data. Because of the relatively few missing values (0.58%), the mean and median imputation was not a distorted representation of the results. Differences between the baseline characteristics of patients in the different tumour location groups were analysed with a one-way ANOVA for continuous variables and a Chi-square test for categorical variables.

The mean values of the CES-D did not differ between the *t*_1a_ and *t*_1b_ time points in patients who were treated with both surgery and radiotherapy; thus, only *t*_1b_ values for patients who had undergone both these assessments were included (*t*_1_).

A linear mixed-effects model with the CES-D score as the outcome was constructed to assess both the changes over time and the effect of the patient characteristics and clinical parameters in patients. To account for within-patient correlations, a random patient factor was added. Fixed-effect factors were assessed, including gender, age, tumour location, tumour size, BMI, education level, living situation, marital status, occupational status, smoking status, alcohol consumption and coping style, as were the two-way interactions of these factors with the assessment period. The factors that were not significant at a *p* < 0.05 level were removed in a backward fashion, beginning with the interactions, to build a parsimonious model with sufficient fit while maintaining a hierarchical structure, meaning that if an interaction was included in the model, the main effects were also represented in the model. The coefficients of the significant covariates, together with the value of the intercept of the mixed model analysis, were combined into a formula for the estimated mean CES-D.

A multiple linear regression model was constructed for the CES-D at 5 years post-treatment. All significant variables from the above-mentioned mixed model were used in a multivariate binary logistic regression model to calculate the probability of depression (defined as CES-D ≥ 16) after 5 years. A receiver operating characteristic (ROC) curve was constructed for this model to facilitate its use in the prediction of depression in our study group. A *p* value < 0.05 was considered statistically significant.

A linear regression with the CES-D score as the outcome was constructed to assess the effect of characteristics in the healthy persons. We considered several potential confounding factors: including gender, age, tumour location, tumour size, BMI, education level, living situation, marital status, occupational status, smoking status, alcohol consumption and coping style. For these variables, we performed unadjusted (i.e. for each variable separately) and adjusted analyses. The unadjusted analysis was performed to explore the potential association for each variable to CES-D. Afterwards, an adjusted model was constructed. Results were reported as regression coefficients with 95% CIs and *p* values. A *p* value of less than 0.05 was accepted as significant.

The mixed model analysis was performed using SAS version 9.4 (SAS institute, Cary, NC, USA). The remaining tests were performed using SPSS 25 (IBM Corp, Armonk, NY, USA).

## Results

**Fig. 1:**
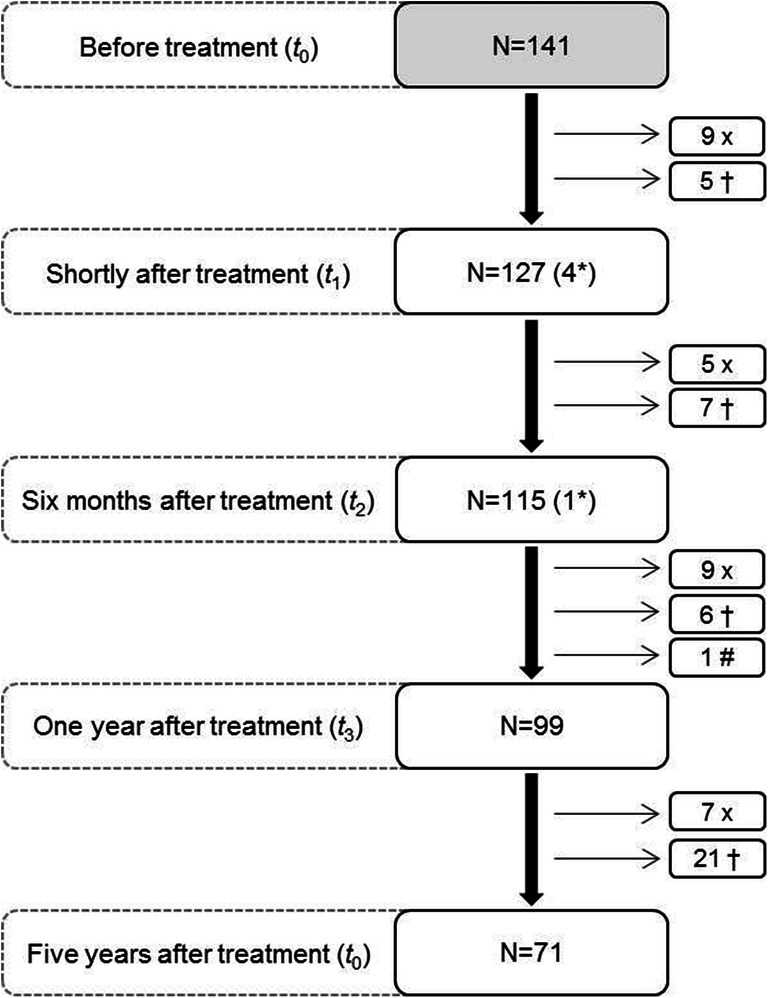
Flowchart showing the number of measurements (*n*) taken at each follow-up timepoint. X, patients stopped participating; †, patients passed away; #, recurrence; *, missing measurement(s)

A total of 141 patients with the mean age of 65.6 (± 12.8) were included in this prospective cohort study, 63 of whom were female (Table 1). Of these, 57 patients had undergone surgery, 20 had received radiotherapy, and 64 had received both surgery and radiotherapy. After 5 years, 71 patients were still participating in this study, 30 patients had stopped participating, 1 patient was excluded from the study because of recurrence of the tumour, and 39 had passed away (see also Fig. 1). Using the threshold CES-D score of 16 as an indicator for clinical depression, 24.8% reported depression before the oncological treatment. At 1 and 5 years after the treatment, the symptoms of depression declined to 20.4% and 17.1%, respectively. For the entire study population, the mean depression score before treatment was 11.4 and declined to 9.3 at 1 year after the treatment and to 8.6 at 5 years after the treatment. The healthy age matched controls reported depression for 11.7%. The mean depression score of the healthy age-matched controls was 10.3.

**Table 1:** Demographic and clinical characteristics of patients and healthy persons

Patient characteristics, *n* (%)	Maxilla (*N* = 34)	Mandible (*N* = 53)	TFM (*N* = 54)	*p* Value	Healthy (*N* = 60)
Sex					
Female	17 (50.0)	25 (47.2)	21 (38.9)	0.534^ǂ^	29 (48.3)
Male	17 (50.0)	28 (52.8)	33 (61.1)		31 (51.7)
Age (years); mean (SD)	68.4 (12.2)	66.6 (12.4)	62.3 (13.0)	0.059^‡^	60.3 (7.2)
BMI; mean (SD)	25.7 (3.8)	25.7 (4.5)	25.4 (4.7)	0.929^‡^	25.2 (3.7)
Treatment					
Surgery	12 (35.3)	23 (43.4)	22 (40.7)	0.702^ǂ^	-
Surgery and radiotherapy	18 (52.9)	24 (45.3)	22 (40.7)		-
Radiotherapy	4 (11.8)	6 (11.3)	10 (18.5)		-
Tumour size (T of TNM)					
T1	5 (14.7)	16 (30.2)	23 (42.6)	0.014*^ǂ^	-
T2	11 (32.4)	13 (24.5)	16 (29.6)		-
T3	1 (2.9)	3 (5.7)	6 (11.1)		-
T4	17 (50.0)	21 (39.6)	9 (16.7)		-
Surgical reconstruction					
No reconstruction	21 (61.8)	22 (41.5)	32 (59.3)	0.000***^ǂ^	-
Local flap	1 (2.9)	2 (3.8)	1 (1.9)		-
Free flap	12 (35.3)	11 (20.8)	19 (35.2)		-
Bone flap	0 (0.0)	18 (34.0)	2 (3.7)		-
Educational level					
Elementary school	8 (23.5)	11 (20.8)	7 (13.0)	0.299^ǂ^	4 (6.7)
Secondary education	18 (53.0)	35 (66.0)	32 (59.3)		33 (55.0)
Higher education; ≥ bachelor	8 (23.5)	7 (13.2)	15 (27.8)		23 (38.3)
Living situation					
Alone with/without children	11 (32.4)	17 (32.1)	16 (29.6)	0.950^ǂ^	3 (5.0)
With partner with/without children	23 (67.6)	36 (67.9)	38 (70.4)		57 (95.0)
Marital status					
Married/living together	23 (67.6)	34 (64.2)	37 (68.5)	0.069^ǂ^	57 (95.0)
Unmarried	2 (5.9)	3 (5.7)	11 (20.4)		2 (3.3)
Divorced	2 (5.9)	2 (3.8)	1 (1.9)		0 (0.0)
Widowed	7 (20.6)	14 (26.4)	5 (9.3)		1 (1.7)
Occupational status					
Paid work	8 (23.5)	12 (22.6)	21 (38.9)	0.129 ^a^	31 (51.7)
No work, retired	26 (76.5)	41 (77.4)	33 (61.1)		29 (48.3)
Smoking (daily)					
Non-smoker	11 (32.4)	12 (22.6)	11 (20.4)	0.770^ǂ^	12 (20.0)
Former smoker	12 (35.3)	20 (37.7)	21 (38.9)		35 (58.3)
Current smoker	11 (32.4)	21 (39.6)	22 (40.7)		13 (21.7)
Alcohol use (daily)					
No more than 1 unit	25 (73.5)	37 (69.8)	30 (55.6)	0.155^ǂ^	30 (50.0)
2 to 4 units	7 (20.6)	13 (24.5)	14 (25.9)		27 (45.0)
More than 5 units	2 (5.9)	3 (5.7)	10 (18.5)		3 (5.0)
Task-orientation coping style; mean (SD)	20.0 (6.2)	20.8 (7.2)	19.1 (6.5)	0.419^‡^	19.4 (5.9)
Emotion-orientation coping style; mean (SD)	10.9 (3.3)	11.1 (4.3)	12.2 (4.9)	0.279^‡^	11.9 (3.9)
Avoidance coping style; mean (SD)	13.1 (3.7)	12.6 (4.1)	13.8 (5.2)	0.341^‡^	14.3 (4.8)
CES-D					
Score < 16	30 (88.2)	40 (75.5)	39 (72.2)	0.201^ǂ^	53 (88.3)
Score ≥ 16	4 (11.8)	13 (24.5)	15 (27.8)		7 (11.7)

*p < 0.05; **p < 0.01; ***p < 0.001; ^ǂ^Chi-square test; ^‡^ANOVA

*BMI* body mass index, *CES-D* Center for Epidemiologic Studies Depression Scale, *TFM* tongue and/or floor of the mouth

### Depression in patients

The mixed-model analysis showed that the type of treatment, tumour size, age, BMI, educational level, living situation, marital level, occupational status, smoking status and alcohol consumption did not significantly contribute to depression; therefore, these factors were removed from the model. Gender, tumour location and having an emotion-oriented coping style did significantly affect the likelihood of a patient experiencing depression, with tumour location and an emotion-oriented coping style influencing depression differently at every assessment. This depression model is depicted in Table 2. Being a female was associated with higher scores on the CES-D depression scale.

**Table 2:** The significant coefficients and interactions derived from the mixed model procedure for depression

	Mixed model	Main effects	SE	Interactions with the assessment moment									
	Intercept	1.399	2.751										
				Before	SE	After	SE	6 months	SE	1 year	SE	5 years	SE
Assessment moment	Before	− 3.022	2.755										
	After	− 0.012	2.776										
	6 Months	− 0.450	2.802										
	1 Year	− 1.882	2.938										
	5 Years	0	0										
Sex	Female	2.657	1.021										
	Male	0	0										
Tumour location	Maxilla	− 2.201	2.120	1.522	2.127	5.771	2.151	7.467	2.197	4.907	2.242	0	0
	Mandible	2.602	1.845	− 1.165	1.855	− 0.413	1.868	0.864	1.888	− 0.410	1.929	0	0
	TFM	0	0	0	0	0	0	0	0	0	0	0	0
Emotion-orientation coping		0.539	0.208	0.454	0.209	0.090	0.211	− 0.062	0.212	0.104	0.227	0	0

Coefficients and SE obtained with the mixed model analysis. Main effect of each independent factor is detailed on the “main effects” row. Significant interactions between factors and assessment moment are shown on the “interactions” row. In order to apply these results on practice, coefficients of categorical values should be multiplied by “1” when present and by “0” when absent. Coefficients of continuous variables should be multiplied by the outcome of that factor

After oncological treatment, patients with a maxillary tumour scored higher on the depression scale than patients with a mandibular tumour. Patients with a tumour on the tongue and/or mouth floor scored lower on the depression scale than patients with a mandibular tumour. The patients with maxilla tumours had the highest increase on the depression scale at 4 to 6 weeks after their oncological treatment, which remained higher until half a year after the treatment. The same pattern is seen for patients with a tumour located at the mandible; however, this pattern is less pronounced. The location of the tumour had less of an impact on the depression scale score at the 1-year and 5-year follow-up assessments, but it is worth mentioning that 5 years after their treatment, patients with a maxilla tumour scored lower for depression than the other patients. A more emotion-orientated coping style resulted in a higher score on the depression scale, which was most pronounced before the intervention.

### Probability of depression 5 years after treatment

**Fig. 2:**
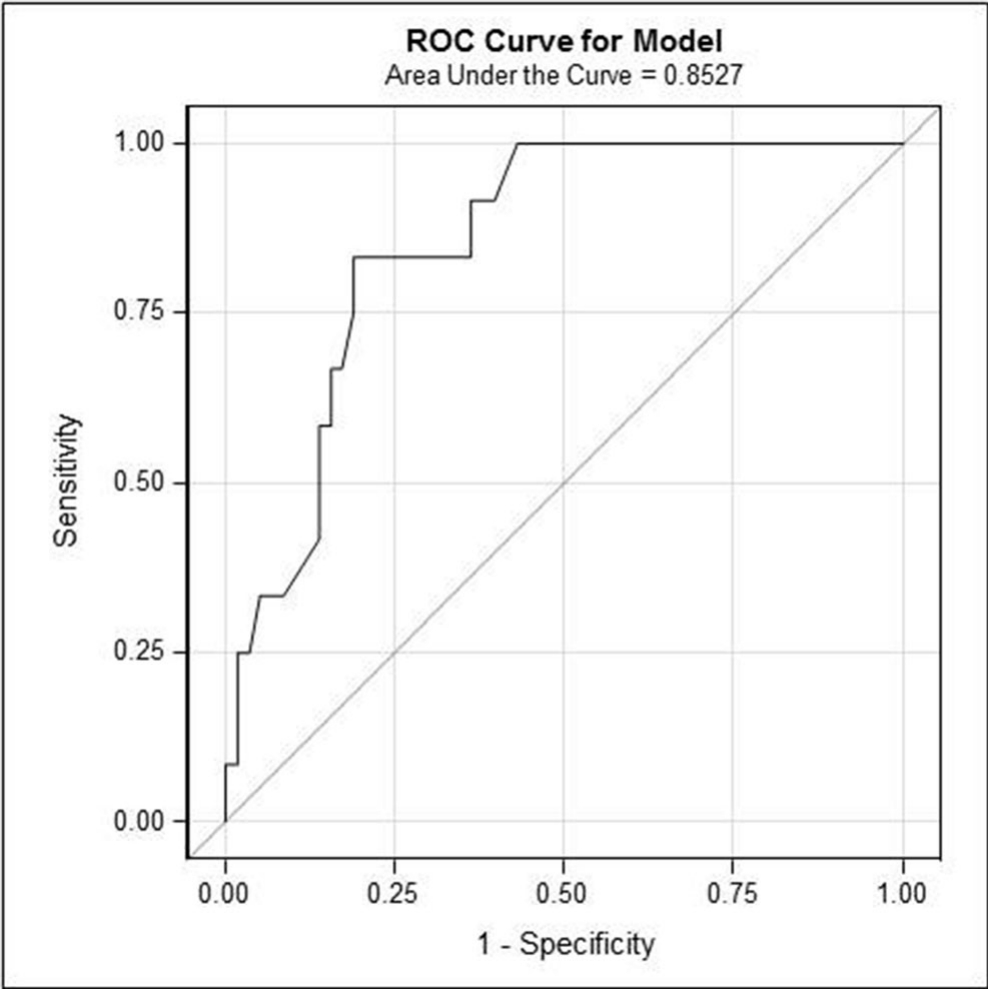
A receiver operating characteristic (ROC) curve for the prediction of depression 5 years after treatment using the logistic regression model (based on gender, tumour location and emotion-oriented coping style). The area under the curve is 0.8297. Areas under the curve can vary between 0 and 1; a value of 0.5 indicates that the model has no diagnostic power, while a 1 indicates that the model has perfect diagnostic accuracy

The combination of gender, tumour location and having an emotion-oriented coping style yielded the largest correlation coefficient in the multiple linear regression model for CES-D at 5 years post-treatment. These three variables were used in a logistical regression to calculate the probability of having a depression 5 years after treatment (Table 3). Having an emotion-oriented coping style (at least 17 points) was the highest risk factor for depression at this time point (1/odds ratio [OR], 0.10204 per point); thus, the probability of developing depression is higher with a strongly emotionally oriented coping style. The other risk factor at the 5-year follow-up appointment was having had a mandibular tumour (OR 3.0). Table 4 summarises the probability of developing depression within 5 years for three patient groups with emotional coping style scores of 7 (P10), 10 (P50) or 17 points (P90). Using this statistical method to predict depression after 5 years yielded an area under ROC of 0.8297 (Fig. 2).

**Table 3:** Logistic regression model for depression five years after treatment

	OR	95% CI	*p* Value
Gender	0.6	0.1 to 2.3	0.416
Tumour location			
TFM	1	N/A	0.350
Maxilla	Non-estimable	-	
Mandible	3.0	(0.7 to 13.1)	
Emotion-oriented coping style			
7	1	N/A	0.017*
10	2.0	(1.1 to 3.5)	
17	9.8	(1.5 to 63.4)	

*p < 0.05; *OR* odds ratio, *N/A* not applicable, *TFM* tongue and/or floor of the mouth

**Table 4:** Probability of depression five years after treatment (based on gender, tumour location and level of emotion-oriented coping style) according to the logistic regression model

Emotion-oriented coping style	Gender	Maxilla		Mandible		TFM	
			95% CI		95% CI		95% CI
7 (P10)	Female	0%	(0–100)	17%	(5–49)	7%	(1–31)
	Male	0%	(0–100)	10%	(3–34)	4%	(1–18)
10 (P50)	Female	0%	(0–100)	30%	(11–59)	12%	(3–39)
	Male	0%	(0–100)	19%	(6–44)	7%	(2–24)
17 (P90)	Female	0%	(0–100)	67%	(31–91)	41%	(14–76)
	Male	0%	(0–100)	53%	(19–85)	28%	(9–61)

*TFM* tongue and/or floor of the mouth

### Depression in healthy persons

In the unadjusted linear regression models, only emotion-oriented coping style was associated with depression (*p* = 0.006; Table 5). Therefore, the adjusted model did not differ from the unadjusted model for emotion-oriented coping style. The effect of emotion-oriented coping style results in an increase of 0.527 points on the CES-D for each point on the emotion-oriented coping style scale (95% CI: 0.154–0.901). So the more the coping style of a person is emotion-oriented, the higher is the level of depression.

**Table 5:** Adjusted and unadjusted linear regression model for depression in healthy persons

Variable	Unadjusted regression coefficients (95% CI)	*p* Value	Adjusted model regression coefficients (95% CI)	*p* Value
Sex				
Female	2.107 (− 0.919–5.133)	0.169		
Male	0			
Age (years)	− 0.049 (− 0.263–0.165)	0.648		
BMI	− 0.218 (− 0.636–0.199)	0.300		
Educational level				
Elementary school	3.848 (−2.548–10.243)	0.233		
Secondary education	− 0.622 (− 3.829–2.585)	0.699		
Higher education; ≥ bachelor degree	0			
Living situation				
Alone with/without children	5.018 (− 1.912–11.947)	0.153		
With partner with/without children	0			
Marital status				
Married/living together	− 1.684 (− 13.542–10.174)	0.777		
Unmarried	5.000 (− 9.397–19.397)	0.490		
Divorced	-	-		
Widowed	0			
Occupational status				
Paid work	− 1.706 (− 4.750–1.337)	0.266		
No work, retired	0			
Smoking (daily)				
Non-smoker	− 1.897 (− 6.653–2.858)	0.428		
Former smoker	− 2.202 (− 6.061–1.656)	0.258		
Current smoker	0			
Alcohol use (daily)				
No more than 1 unit	− 6.933 (− 13.935–0.068)	0.052		
2 to 4 units	− 5.111 (− 12.148–1.926)	0.151		
More than 5 units	0			
Task-orientation coping style	0.170 (− 0.089–0.428)	0.193		
Emotion-orientation coping style	0.527 (0.154–0.901)	0.006**	0.527 (0.154–0.901)	0.006**
Avoidance coping style	0.178 (− 0.141–0.498)	0.268		
Intercept			4.281 (− 0.400–8.961)	
*R*^2^				0.121

**p < 0.01; *CI* confidence interval. The unadjusted model showed the association of each variable to depression. The adjusted model showed depression and emotion-orientation coping style of healthy persons (*R*^2^ = 0.121)

## Discussion

Gender, tumour location and having an emotion-oriented coping style were identified as characteristics involving depression in patients treated for oral cancer. At 1 and 5 years after the treatment, the prevalence of depression decreased. The occurrence of depression (defined as CES-D ≥ 16) within 5 years after treatment could be reliably predicted by a patient’s gender, the location of their tumour and the extent to which they had an emotion-oriented coping style. Emotion-oriented coping style was also identified as characteristic involving depression in the healthy age-matched controls.

Of the healthy age-matched controls, 11.7% had depression (CES-D ≥ 16), while 24.8% of the patients with oral cancer reported having depression before their oncological treatment, and 20.4% and 17.1%, respectively, had depression at 1 and 5 years after the treatment. This revealed that patients with oral cancer had a higher prevalence of depression than the healthy controls, although this did decrease over time after their treatment. In the Longitudinal Aging Study Amsterdam, it was shown that persons between 64 and 84 years had a depression prevalence of 12.5% to 16.6%, which is also less than the prevalence’s we found before and up to 1 year after oral cancer treatment [36]. Thereby, the levels of depressive symptoms in our study were consistent with the results of other studies on depression rates in patients with cancer; for example, the published literature also showed that the mean depression score for the population of patients with cancer rises 3 to 6 months after treatment and starts to decrease 1 year after treatment [37]. Currently, no published studies have determined the depression risk for patients at 5 years after oral cancer treatment. In clinic we experience that patients who survived oral cancer get used to their remaining deficits and are becoming less afraid of dying from cancer. Patients may therefore show a decreasing depression rate starting at 1 year after their oncological intervention.

Here, we showed that patients treated for a maxilla tumour were significantly more likely to develop depression shortly after treatment than the other patients. Shortly after treatment, patients requiring an obturator prosthesis often do not have yet their final prostheses, which can limit speech [38], eating [39] and influence the mood of the patients.

This study showed that female patients had a significantly higher risk for clinical depression, which is in line with the current literature [40]. This finding might have resulted from biological factors (such as hormones) and social factors; for example, the experience of stress may be different for females than males [41], but also disfigurement may be different for females than males [42]. On the other hand, being a male is a protective factor for clinical depression based on the results of this study.

In addition, this study showed that a higher emotion-oriented coping style significantly increases the risk of developing depression in both patients and healthy age-matched controls. According to the current literature, increases in depression symptoms are related to a person’s preference for emotion-oriented rather than task-oriented coping strategies [43], which was consistent with our findings.

Two of the cohort study’s strengths are the longitudinal design and the implementation of the 5-year post-treatment follow-up assessment. No previous study has presented the depression rates of oral cancer patients 5 years after treatment. We included a broad range of patient characteristics and coping strategies that could potentially predict the depressive symptoms of patients before treatment to 5 years after treatment. Moreover, another strength of the cohort study is the generalisability of the study population. The patients were recruited from two of the eight academic head and neck cancer centres in the Netherlands. To fully appreciate the present results, some additional points must be considered. First, given that this study has no experimental aspect, it is not clear whether the patient was treated for possible depressive symptoms by, for example, their general practitioner, or if any other interventions were used to prevent or treat depressive symptoms. In addition, the depression scale, coping questionnaire and patient characteristics were self-reported, which could have resulted in underreported effects [44]. Furthermore, the CISS-21 questionnaire is a shorter version of the validated CISS-48 questionnaire and has not been completely validated in its own right.

The findings of this study could be used to enable nurses to recognise patients with oral cancer who are at a higher risk for depression, allowing them to enact measures to prevent them from developing severe depression. These results could also be used to optimise the counselling treatments provided by nurses, which has been proven to be effective at reducing depressive symptoms by providing information about the use of effective coping styles [45]. It is therefore essential to train nurses in how to accurately use depression-screening instruments.

Future prospective studies should test whether the number of depressive symptoms (CES-D scores) is related to the quality of oral functioning, the QoL and the amount of disfigurement. It is also important to consider whether the type of nursing intervention fits with the counselling of different patient characteristics that predict depression. Thus, this study should be repeated while implementing a validated coping questionnaire, which would provide nurses with effective information about the coping styles used by patients with oral cancer.

In conclusion, this study revealed that being female, having a maxillary tumour and having an emotion-oriented coping style are associated with higher levels of depressive symptoms in patients treated for oral cancer up to 5 years post-treatment. A substantial proportion of the patients with oral cancer experienced high levels of depression both before and after their treatment, suggesting that adequate diagnostics and care are needed to try to prevent severe depression in these patients. In healthy persons an emotion-oriented coping style is also associated with higher levels of depressive symptoms; however, the effect is smaller than in patients treated for oral cancer.

## Data Availability

The authors have full control of all primary data and agree to allow the journal view primary data upon request.
